# Vimentin expression in circulating tumor cells (CTCs) associated with liver metastases predicts poor progression-free survival in patients with advanced lung cancer

**DOI:** 10.1007/s00432-019-03040-9

**Published:** 2019-10-23

**Authors:** Ying Wang, Yanxia Liu, Lina Zhang, Li Tong, Yuan Gao, Fanbin Hu, Peter Ping Lin, Baolan Li, Tongmei Zhang

**Affiliations:** 1 Department of Cellular and Molecular Biology, Beijing Tuberculosis and Thoracic Tumor Research Institute, Beijing Chest Hospital, Capital Medical University, No.9, Beiguan Street, Tongzhou District, Beijing, 101149 China; 2 Department of Oncology, Shijingshan Teaching Hospital of Capital Medical University, Beijing Shijingshan Hospital, Beijing, 100043 China; 3 Department of Oncology, Beijing Tuberculosis and Thoracic Tumor Research Institute, Beijing Chest Hospital, Capital Medical University, No.9, Beiguan Street, Tongzhou District, Beijing, 101149 China; 4 Department of General Medicine, Beijing Tuberculosis and Thoracic Tumor Research Institute, Beijing Chest Hospital, Capital Medical University, No.9, Beiguan Street, Tongzhou District, Beijing, 101149 China; 5 Cytelligen, San Diego, CA USA

**Keywords:** Vimentin, Circulating tumor cells, Cell size, Aneuploidy of Chr8, Advanced lung cancer, Progression-free survival

## Abstract

**Objective:**

To investigate the presence of vimentin expression in CTCs and its clinical relevance in patients with advanced lung cancer.

**Methods:**

Peripheral blood was obtained from 61 treatment-naive patients with advanced lung cancer. Subtraction enrichment and immunostaining-fluorescence in situ hybridization (SE-iFISH) platform was applied to identify, enumerate and characterize CTCs based on cell size, aneuploidy of chromosome 8 (Chr8) and vimentin expression. Quantification and analysis of CTCs were performed on patients before chemotherapy administration and after two cycles of therapy.

**Results:**

Before treatment, CTCs were detected in 60 (98.4%) patients, small cell CTCs (≤ 5 µm of WBCs) accounted for 52.8% of the absolute CTCs number, while 12 (19.7%) of the included patients had detectable vimentin-positive CTCs (vim^+^ CTCs). Liver metastases were reported in 7 (11.5%) patients and were significantly correlated to the presence of Vim^+^ CTCs (*p *= 0.002), with a high positivity rate of 71.4% (5/7). Vim^+^ CTCs were mostly in small cell size and Chr8 aneuploidy (77.0% and 82.05%, respectively). Baseline small cell CTCs ≥ 2/6 ml, triploid CTCs ≥ 2/6 ml, Vim^+^ CTCs ≥ 1/6 ml were found to significantly correlate with poor progression-free survival (PFS) (*p *= 0.017, *p *= 0.009 and *p *= 0.001, respectively). After adjusting for clinically significant factors, baseline Vim^+^ CTCs ≥ 1/6 ml was the only independent predictor of poor PFS [hazard ratio (HR):2.756, 95% confidence interval (CI): 1.239–6.131; *p *= 0.013].

**Conclusions:**

This study demonstrates an important morphologic, karyotypic and phenotypic CTCs heterogeneity in advanced lung cancer patients. The majority of Vim^+^ CTCs are in small size and Chr8 aneuploidy. Baseline presence of Vim^+^ CTCs is correlated with liver metastases and may help predict poor PFS.

**Electronic supplementary material:**

The online version of this article (10.1007/s00432-019-03040-9) contains supplementary material, which is available to authorized users.

## Introduction

Lung cancer remains a leading cause of cancer death worldwide, approximately 1.8 million new cases and 1.6 million lung cancer deaths occured every year globally, among which a third occured in China (Chen et al. 2016; Ferlay et al. 2015). The high mortality of cancer patients is in most cases caused by metastatic disease but the underlying mechanisms regarding this complex process are incompletely elucidated (Wan et al. 2013). Primary carcinomas are known to constantly shed tumor cells into the bloodstream, after cell subpopulations have presumably undergone an phenotype of epithelial to mesenchymal transition (EMT) reversion (Nieto et al. 2016). EMT endows tumor cells with invasive and metastatic abilities which in effect allow cells to penetrate into the lymph vasculature and circulate as single or clusters of CTCs (Nurwidya et al. 2016), whilst CTCs exist in a dynamic EMT state in blood (Micalizzi et al. 2017). There is evidence that EMT is critical for both metastasis and therapeutic resistance and vimentin is regarded as one of the best indicators of EMT in tumorigenesis (Michael and Neilson 2009; Thiery 2002).

Human malignant carcinomas, including lung cancer, are highly heterogeneous, and their molecular characteristics evolve to induce metastasis and therapeutic resistance during disease progression (Amirouchene-Angelozzi et al. 2017; Maley et al. 2017; Messaritakis et al. 2018; Nicholas and Charles 2015). Analysis of CTCs, which are homologous with the primary or metastatic solid tumors, offers an ideal approach to real-time monitor the tumor heterogeneity (Stott et al. 2010). Previous studies have revealed the clinical significance of CTCs count as well as their dynamic change in patients with lung cancer (Hayes et al. 2006; Hiltermann et al. 2012; Jian-Mei et al. 2012; Normanno et al. 2014). In addition to enumerating CTCs, to directly pinpoint specific CTC subpopulations characterized by cell size, chromosome ploidy and tumor biomarkers may help guide more precise personal therapy and allow more robust analyses. Accurate identification and analysis of Vim^+^ CTCs, which represent specific phenotype of EMT, are of great importance in comprehensive understanding their biological functions and clinical significance. Taking advantages of the well-established integrated SE-iFISH platform, CTCs with different morphologic, karyotypic and phenotypic characteristics can be efficiently isolated and effectively identified (Li et al. 2018; Lin 2018).

Since vimentin has been considered to be a critical prerequisite for tumor metastasis, we prospectively designed this study to investigate the presence of vimentin expression in CTCs and whether specific CTC subpopulations and their dynamic changes could serve as biomarkers associated with treatment response and prognosis in patients with advanced lung cancer.

## Patients and methods

### Patient enrollment and sample collection

This is a prospective non-interventional follow-up study. From December 2017 to December 2018, 61 newly diagnosed patients (≥ 18 years) with inoperable, locally advanced (Stage IIIA/IIIB) or metastatic (Stage IV) lung cancer were recruited in the study at Beijing Chest Hospital, Capital Medical University. Among those patients, 44 cases of small cell lung cancer (SCLC) and 17 cases of driver gene-negative adenocarcinoma (ADC) were histopathologically diagnosed and genetically validated. Patients who had not received treatment and those who had a performance status (PS) ≥ 2, with adequate organ function and evaluable tumor lesions were eligible for this study. Patients with a history of other malignant tumors were excluded. SCLC patients received the first-line platinum-based chemotherapy alone or plus concurrent or sequential radiotherapy, while ADC patients received the first-line bevacizumab targeted therapy plus platinum-based chemotherapy. Six weeks (two cycles) after treatment initiation, evaluation of clinical response was performed using computed tomography (CT) according to Response Evaluation Criteria in Solid Tumors (RECIST) version 1.1 criteria. Responses were categorized as partial response (PR, at least a 30% decrease in the sum of diameters of target lesions), progressive disease (PD, at least a 20% increase in the sum of diameters of target lesions), or stable disease (SD, neither sufficient shrinkage to qualify as PR nor sufficient increase to qualify as PD). 6 ml of peripheral blood was drawn prior to chemotherapy administration (*n* = 61) and after two cycles of therapy (*n* = 59). All experiments were performed within 48 h after peripheral blood sample collection and result slides were collected and stored at room temperature in the dark. The evaluation of CTCs was done blindly to clinical data. Results are expressed as number of CTCs/6 ml blood. This study was conducted according to the principles of the Declaration of Helsinki and approved by the ethical committees of the Beijing Chest Hospital, Capital Medical University. All participants signed written informed consent.

### Subtraction enrichment of CTCs

Subtraction enrichment experiment was performed according to the kit instructions (Cytelligen, San Diego, CA, USA) and the protocols similarly to that previously published with certain modifications (Li et al. 2018; Ye et al. 2019). Briefly, 6 ml of peripheral blood of patients with advanced lung cancer were collected into a tube containing Acid Citrate Dextrose (ACD) anti-coagulant (Becton–Dickinson, Franklin Lakes, NJ) after discarding the first 2 ml of blood to avoid epithelial cell contamination. Blood samples were centrifuged at 200×*g* for 15 min at room temperature. Sedimented blood cells were gently mixed with 3 ml hCTC buffer, followed by loading on the non-hematopoietic cell separation matrix in a 50-ml tube, and subsequent centrifugation at 450×*g* for 6 min. The middle layer containing white blood cells (WBCs) and tumor cells, but not red blood cells (RBCs) was collected into a 50-ml tube and subsequently incubated with 300 μl of anti-CD45 monoclonal antibody-coated magnetic beads at room temperature for 20 min with gentle shaking. WBCs bound to magnetic beads were separated using a magnetic frame (Promega, Madison, WI). The bead-free supernatants were transferred into a 15-ml tube, followed by adding hCTC buffer to 14 ml. Samples were spun at 500×*g* for 4 min at room temperature. Supernatants were aspirated down to 50 μl. Sedimented cells in 50 μl solution were gently resuspended, followed by mixing with the special fixative produced by Cytelligen, then applied to the coated and formatted CTC slides. Cell pellet was dried overnight at 37 °C for subsequent iFISH analyses.

### Vimentin-iFISH

Vimentin-iFISH was performed similarly to that previously published (Li et al. 2018), and according to the kit instructions (Cytelligen). Briefly, dried monolayer cells on the CTC slides were rinsed and incubated with PBS at room temperature for 3 min, followed by hybridization with Chr8 centromere probe (CEP8) Spectrum Orange (Vysis, Abbott Laboratories, Abbott Park, IL) using a S500 StatSpin Thermo Brite Slide Hybridization/Denaturation System (Abbott Molecular, Des Plaines, IL, USA). Samples were subsequently incubated with Alexa Fluor (AF) Cy7 (pink) and 594 (red), respectively, conjugated to the mAbs representing vimentin and CD45 at room temperature for 20 min in the dark. After washing, samples were covered with mounting media containing 4′,6-diamidino-2-phenylindole (DAPI) for nucleus staining (Vector Laboratories, Burlington, CA), and subjected to automated CTC image scanning and analyses.

### Automated CTC scanning and image analysis performed by Metafer-iFISH^®^

Metafer-iFISH^®^, an automated scanning and image analyzing system (Carl Zeiss, Oberkochen, Germany; MetaSystems, Altlussheim, Germany; and Cytelligen, San Diego, CA, USA) was applied to finish scanning, image acquiring and analysis of positive iFISHed CTCs on the slides (Li et al. 2018). Briefly, every sample slide automatically loaded on a Zeiss fluorescence microscope (AXIO Imager. Z2) was subjected to automated full X–Y plane scanning with cross Z-sectioning of all cells performed at 1-μm step width depth, to acquire entire fluorescence signal of each multicolor channel. Automated CTCs classification and statistical analysis were performed upon cell size, chromosome ploidy and immunostaining intensity of vimentin expression. CTCs are identified as DAPI^+^/CD45^−^/Vim^+/−^ with aneuploid Chr8 or DAPI^+^/CD45^−^/Vim^+^ with diploid Chr8. Small cell CTCs are defined as the maximum diameter of CTCs smaller than 5 µm while large cell CTCs are defined as the maximum diameter of CTCs larger than 5 µm. The precise copy number of Chr8 was assessed in every single CTCs. All sample slides were independently reviewed by two skilled investigators.

### Statistical analysis

All statistical analyses were conducted using SPSS 21.0 software (Chicago, IL, USA). Due to the small sample size of this study, median numbers of total CTCs and diverse CTC subpopulations were used as cut-off points. For Vim^+^ CTCs which the median number was 0, 1was applied as the cut-off point. Chi-square test and Fisher’s exact test were used to compare categorical data. Continuous data were expressed as median and interquartile range (IQR) where appropriate. Comparisons of continuous variables between the two groups were performed using the Mann–Whitney test. Kaplan–Meier survival plots for PFS were generated based on different CTC subpopulations, and the survival curves were compared using log-rank test. Univariate and multivariate Cox proportional hazards regression models with HR and 95% CIs were used to determine the association between potential prognostic factors and PFS. CTC subpopulations as well as standard clinical factors were subjected to univariate analysis for PFS. Significant variables from univariate analysis were included in multivariate analysis. *p* < 0.05 was considered statistically significant.

## Results

### Detection of total CTCs in advanced lung cancer patients

The clinical characteristics of the 61 enrolled patients are listed in Table 1. Quantification of CTCs in pre-treatment patients was performed and results revealed the positivity of CTCs in 60 out of 61 (98.4%) patients, the median CTC count was 5/6 ml (IQR 2–11). Correlation of CTCs in pretreatment patients with clinical characteristics was investigated. Based on the SE-iFISH results, a higher (≥ 5/6 ml) number of CTCs was detected in 33 (54.1%) patients. The detection of CTCs ≥ 5/6 ml was significantly associated with patients’ smoking history (*p *= 0.008) and liver metastases (*p *= 0.029) but not pathological type and other clinical factors (Table 1). Quantitative comparison of CTCs in patients with different pathological types revealed no obvious difference between SCLC and ADC patients (data not shown).

**Table 1:** Correlation of total CTCs and Vim^+^ CTCs in pre-treatment patients with clinical characteristics (n = 61)

Characteristics	*n* (%)	Total CTCs		*P*	Vim^+^ CTCs		*P*
		≥ 5	< 5		≥ 1	< 1	
Age							
≥ 60	38 (62)	21	17	0.814	7	31	0.752
< 60	23 (38)	12	11		5	18	
Gender							
Male	51 (84)	25	26	0.147	11	40	0.684
Female	10 (16)	8	2		1	9	
PS							
0	8 (13)	4	4	0.856	1	7	0.944
1–2	53 (87)	29	24		11	42	
Smoking history							
Yes	52 (85)	24	28	0.008	11	41	0.806
No	9 (15)	9	0		1	8	
Histology type							
SCLC	44 (72)	23	21	0.645	9	35	0.911
ADC	17 (28)	10	7		3	14	
TNM stage							
III	25 (41)	13	12	0.784	3	22	0.353
IV	36 (59)	20	16		9	27	
Liver metastasis							
Yes	7 (11)	7	0	0.029	5	2	0.002
No	54 (89)	26	28		7	47	
Bone metastasis							
Yes	8 (13)	4	4	0.896	1	7	0.944
No	53 (87)	29	24		11	42	
Brain metastasis							
Yes	3 (5)	2	1	0.884	1	2	0.893
No	58 (95)	31	27		11	47	
Treatment response							
PR	37 (61)	21	16	0.605	7	30	0.854
SD + PD	24 (39)	12	12		5	19	

Finally, an objective response was achieved in 37 (60.7%) patients while 13 (21.3%) and 11 (18.0%) patients experienced stable or progressive disease, respectively. We, therefore, evaluated the association between dynamic change of CTCs and clinical efficiency. Result revealed the change of total CTCs before and after treatment was significantly correlated with treatment response (*p *= 0.024) (Suppl Table 1).

### Analysis of CTCs based on cell size and correlation of small cell CTCs with PFS

**Fig. 1:**
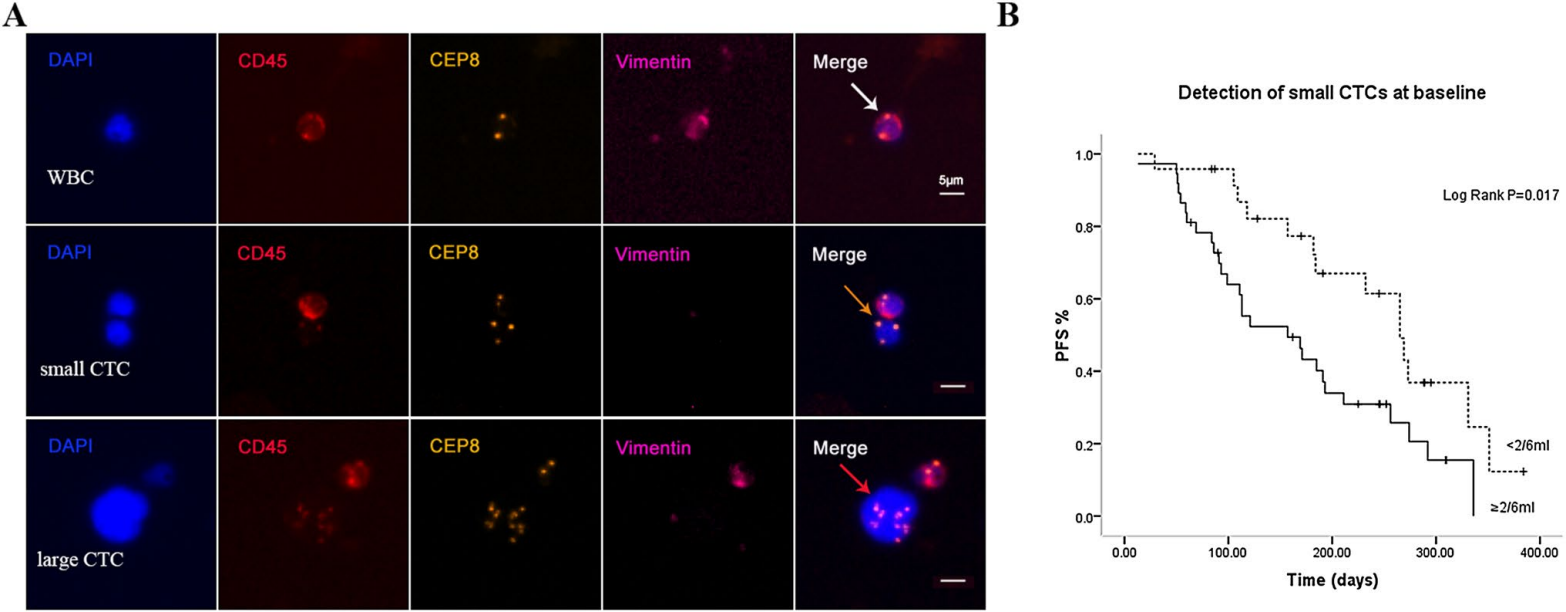
Analysis of CTCs based on cell size and correlation of small cell CTCs with PFS. **a** Images of small and large CTCs in advanced lung cancer patients. Comparing to the size of WBCs (white arrow), an small (≤ 5um WBC) triploid CTCs as well as an large (≥ 5um WBC) multiploid CTCs are indicated by orange and red arrow. **b** Correlation of small cell CTCs with PFS. Pretreatment patients with small cell CTCs ≥ 2/6 ml had a PFS significantly shorter than patients with small cell CTCs < 2/6 ml (*p *= 0.017)

The cell size of CTCs was commonly larger than hematopoietic cells (Vona et al. 2000a). Our study revealed the diameter of CTCs ranged from 2–4 to 20–30 µm. Based on cell size, CTCs were categorised as small cell CTCs (Fig. 1a, orange arrows) and large cell CTCs (Fig. 1a, red arrows). Obtained results showed that pretreatment small cell CTCs accounted for 52.8% of the absolute CTCs number and the median count was 2/6 ml (IQR 1–6). No obvious variation in terms of ratio of small cell CTCs was observed in advanced lung cancer patients before and after treatment (52.8% vs. 53.6%).

With respect to the clinical significance of small cell CTCs, we found the decrease of small cell CTCs during treatment was significantly associated with better treatment response (*p *= 0.026) (Suppl Table 1). Depicted in Fig. 1b, pretreatment patients with small cell CTCs ≥ 2/6 ml had a PFS significantly shorter than patients with small cell CTCs < 2/6 ml (*p *= 0.017). However, no similar result was obtained in post-treatment small cell CTCs.

### Analysis of Chr 8 ploidy of CTCs and correlation of triploid CTCs with PFS

**Fig. 2:**
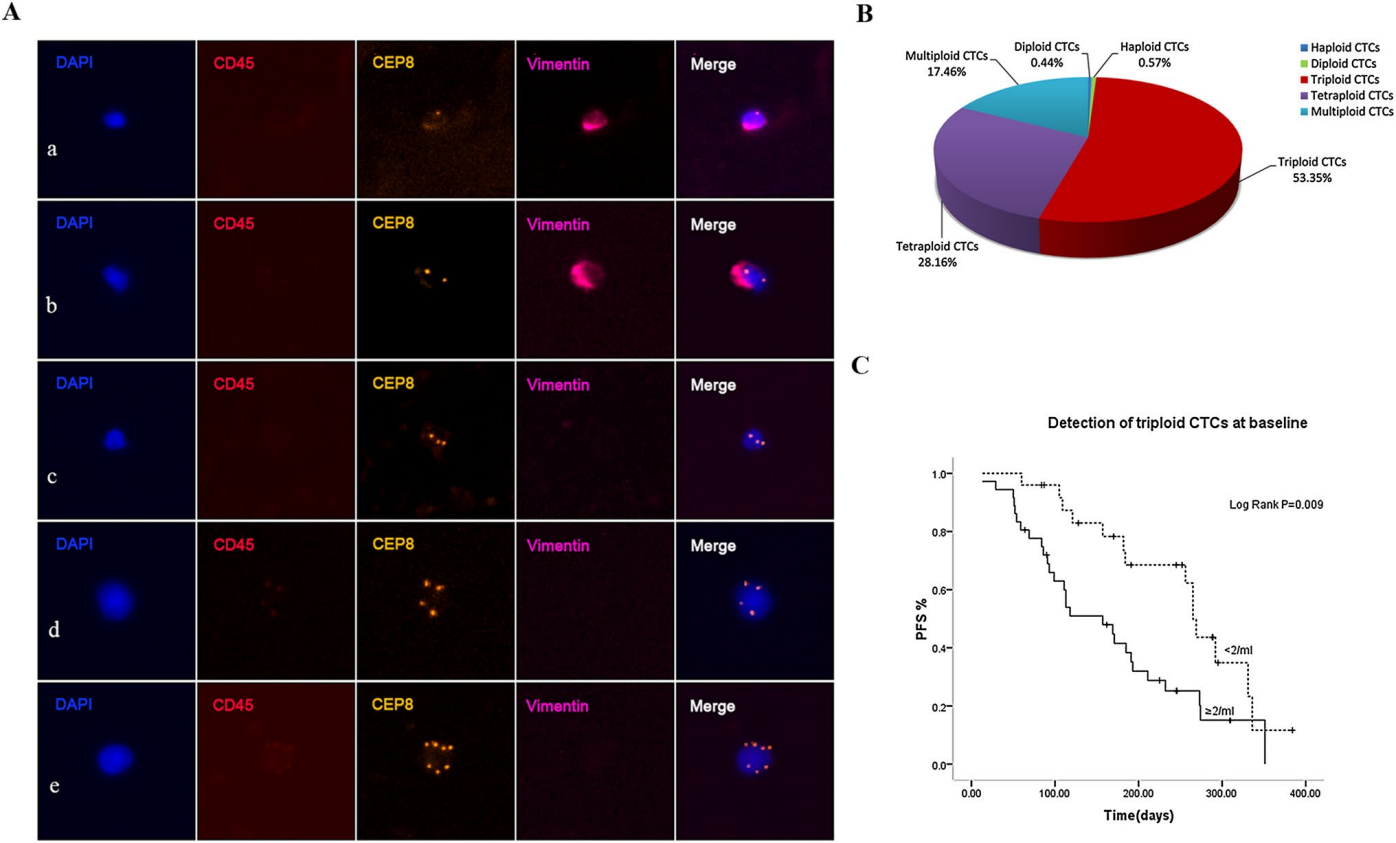
Analysis of Chr 8 ploidy of CTCs and correlation of triploid CTCs with PFS. **A** Images of diverse Chr8 ploidy CTCs. a Haploidy, b diploidy, c triploidy, d tetraploidy, e multiploidy copies. **B** Proportion of diverse Chr8 ploidy CTCs in pretreatment patients. **C** Correlation of triploid CTCs with PFS. Baseline triploid CTCs ≥ 2/6 ml showed a much shorter PFS than patients with triploid CTCs < 2/6 ml (*p *= 0.009)

SE-iFISH revealed an important CTCs heterogeneity based on karyotyping of Chr8 ploidy (haploid, diploid, triploid, tetraploid, multiploid CTCs) in advanced lung cancer patients Fig. 2A. As shown in Fig. 2B, before treatment, more than half of the total detected CTCs were triploid CTCs, with a proportion of 53.35% and a median count of 2/6 ml (IQR 0.5–4). The second to the largest of CTC subpopulations were tetraploid CTCs with a proportion of 28.16% followed by multiploid CTCs which accounted for 17.46% of the entire CTCs number.

Depicted in Fig. 2C, pretreatment triploid CTCs ≥ 2/6 ml showed a much shorter PFS, indicating that triploid CTCs significantly associated with patients’ poor prognosis (*p *= 0.009). However, post-treatment triploid CTCs were not found to significantly correlated with PFS in our study (data not shown).

### Detection of vimentin expression in Chr8 aneuploid CTCs and correlation of Vim^+^ CTCs with PFS

At baseline, SE-iFISH revealed the presence of Vim^+^ CTCs in 12 (19.7%) out of 61 patients (Table 1). As for the quantitative composition, Vim^+^ CTCs accounted for 2.5% of the absolute CTCs number in pretreatment patients with a median count of 0/6 ml (range 0–20). Obtained results revealed the majority of Vim^+^ CTCs were in small size, which accounted for 77.04% of them and there was significant difference in cell size between Vim^+^ CTCs and Vim^−^ CTCs (*p* < 0.001).

**Fig. 3:**
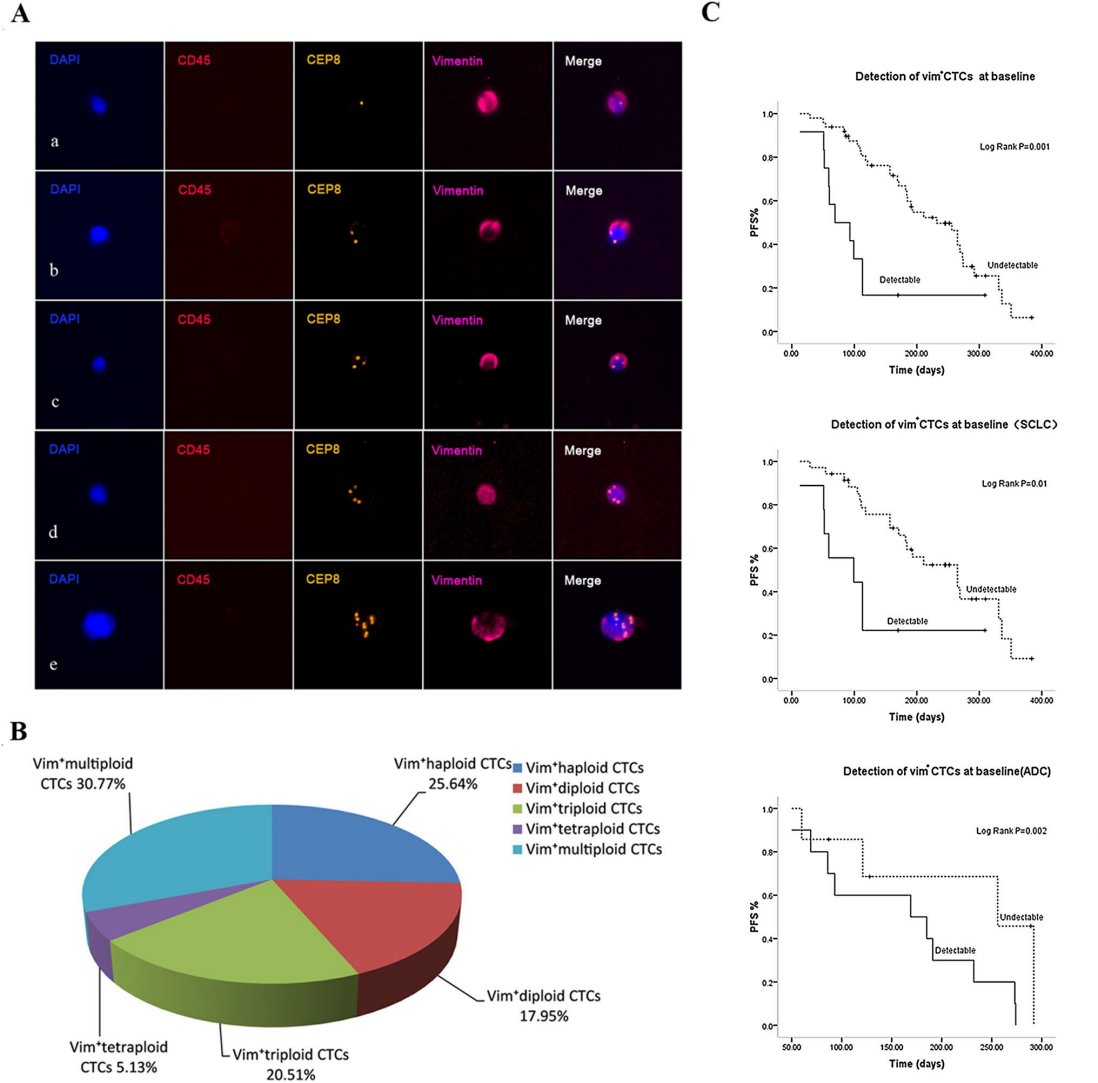
Detection of vimentin expression in diverse Chr8 ploidy CTCs and correlation of Vim^+^ CTCs with PFS. **A** Vimentin expression in diverse Chr8 ploidy CTCs. a–e Vim^+^ CTCs with haploid, diploid, triploid, tetraploid, and multiploid Chr8, respectively. **B** Proportion of diverse Chr8 ploidy Vim^+^ CTCs in pretreatment patients. **C** Correlation of Vim^+^ CTCs with PFS. Patients with advanced lung cancer showing poor prognosis had positive detection of Vim^+^ CTCs (≥ 1/6 ml). Subgroup analysis showed the presence of Vim^+^ CTCs indicating worse prognosis in both SCLC and ADC patients

Additional investigation was performed on Vim^+^ CTCs based on Chr8 ploidy (Fig. 3A). Among those Vim^+^ CTCs, the ratios of haploid, diploid, triploid, tetraploid and multiploid CTCs were 25.64%, 17.95%, 20.51%, 5.13% and 30.77%, respectively, which indicated more than 80% of Vim^+^ CTCs were Chr8 aneuploid (Fig. 3B). Correlation analysis showed significant association between the presence of Vim^+^ CTCs and liver metastases (*p *= 0.005) (Table 1).

Analysis of pretreatment Vim^+^ CTCs was depicted in Fig. 3C, only those patients showing poor prognosis had positive detection of Vim^+^ CTCs (≥ 1/6 ml) (*p *= 0.001). To fully investigate the prognostic value of Vim^+^ CTCs in different pathological types, subgroup analysis was conducted as well. Results revealed the positive detection of Vim^+^ CTCs indicated worse prognosis in both SCLC and ADC patients (*p *= 0.01, *p *= 0.002). Post-treatment Vim^+^ CTCs did not show significant correlation with PFS in our study.

### Univariate and multivariate analysis

Up to the last follow-up time, 42 patients experienced disease progression. The median PFS was 170 days. Univariate analysis of CTC subpopulations revealed that pretreatment small cell CTCs ≥ 2/6 ml, triploid CTCs ≥ 2/6 ml and Vim^+^ CTCs ≥ 1/6 ml were significantly associated with a shorter PFS (*p *= 0.017, *p *= 0.009 and *p *= 0.001, respectively). Correlation of standard clinical factors with disease progression was also evaluated. As expected, two clinical factors including disease stage and liver metastases showed statistical significance in univariate analysis (*p *= 0.012, *p *= 0.001). To further explore prognostic factors affecting PFS, factors that were significant in univariate analysis were entered into the multivariate Cox proportional hazard model. Pretreatment Vim^+^ CTCs and TNM stage emerged as independent factors associated with PFS [HR: 2.349, 95% CI: (1.128–4.889), *p *= 0.022; HR: 2.756, 95% CI: (1.239–6.131), *p *= 0.013, respectively; Table 2].

**Table 2:** Univariate and multivariate analysis for prognosis predictors in advanced lung cancer patients

Variables	Univariate analysis		Multivariate analysis	
	HR(95% CI)	*p*	HR(95% CI)	*p*
Age ≥ 60 vs. < 60	0.956 (0.399–2.291)	0.919		
Gender male vs. female	0.757 (0.407–1.408)	0.757		
PS 1–2 vs. 0	2.701 (0.947–7.702)	0.063		
Smoking history yes vs. no	1.021 (0.425–2.452)	0.963		
Pathological type SCLC vs. ADC	0.674 (0.350–1.298)	0.238		
TNM stage III vs. VI	2.322 (1.182–4.561)	0.014	2.349 (1.128-4.889)	0.022
Liver metastasis yes vs. no	3.923 (1.593–9.662)	0.003	1.641 (0.602–4.474)	0.333
Bone metastasis yes vs. no	1.033 (0.403–2.650)	0.946		
Brain metastases yes vs. no	0.935 (0.223–3.912)	0.927		
Treatment response PR vs. SD + PD	1.762 (0.941–3.299)	0.077		
Baseline CTCs ≥ 5 vs. < 5	1.119 (0.607–2.066)	0.718		
Baseline small cell CTCs ≥ 2 vs. < 2	2.208 (1.133–4.303)	0.020	1.262 (0.580–2.747)	0.558
Baseline triploid CTCs ≥ 2 vs. < 2	2.310 (1.206–4.424)	0.012	1.764 (0.827–3.579)	0.142
Baseline Vim^+^ CTCs ≥ 1 vs. 0	3.358 (1.600–7.049)	0.001	2.756 (1.239–6.131)	0.013

## Discussion

The clinical significance of CTCs in various carcinomas has been well established by previous studies (Hayes et al. 2006; Hiltermann et al. 2012; Jian-Mei et al. 2012; Normanno et al. 2014; Stott et al. 2010). However, the rarity and molecular and phenotypical heterogeneity of CTCs make it rather difficult to isolate and identify them efficiently. Conventional CTCs detection technologies mainly rely on EpCAM-dependent capture, tumor cell size-based filtration for isolation, and immunostaining of intracellular CKs for identification (Lin 2015; Vona et al. 2000b; Yu et al. 2011). However, dynamic heterogeneity, which may result in complete absence of the anchor proteins targeted for detection and change of cell size lead to a considerable amount of CTCs “uncapturable” and “invisible”. In our study, SE-iFISH platform was applied to enrich CTCs with high efficiency, followed by in situ morphologic, karyotypic and phenotypic analyses of CTCs based on cell size, Chr8 aneuploidy and immunostaining of vimentin. In the current study, SE-iFISH demonstrated a comparable higher positive rate of 98.4% than detection sensitivity documented by conventional technologies that are relying on EpCAM expression (Messaritakis et al. 2017; Xu et al. 2015). Correlation analysis of CTCs to clinical characteristics showed a higher total CTCs number (≥ 5/6 ml) was significantly associated with patients’ smoking history and liver metastases.

Small cell CTCs which may escape from filtration detection by cell size based isolation technology, accounted for more than half of the absolute CTCs number in pretreatment patients by SE-iFISH platform in our study. Extensive investigation regarding to the clinical significance of small cell CTCs was also evaluated in current study. According to the obtained results, the dynamic change of small cell CTCs number before and after treatment was demonstrated correlated to treatment response. Moreover, pretreatment small cell CTCs (≥ 2/6 ml) were highly related to poor prognosis in advanced lung cancer patients. Though previous studies have documented the mesenchymal CTCs or CTCs in patients following therapeutic treatment were in small cell size (Hiroaki et al. 2014; Ito et al. 2016), few studies have been conducted to further investigate how diverse CTCs based on cell size correlate to clinical outcome due to the inherent drawbacks of the conventional CTCs technology. As far as we know, it is the first time in our study to report the dynamic change of small cell CTCs number correlated to treatment response and pretreatment small cell CTCs (≥ 2/6 ml) were significantly related to unfavorable clinical outcome in advanced lung cancer patients.

In view of the overexpression of EMT related protein vimentin as well as Chr8 aneuploidy in tumor cells predict poor prognosis in many carcinomas including lung cancers (Du et al. 2018; Karim et al. 2017), both karyotypic and phenotypic of CTCs from patients with newly diagnosed advanced lung cancer were evaluated by SE-iFISH platform in current prospective study. Karyotyping revealed that triploid CTCs accounted for the largest proportion of the entire CTCs in pretreatment patients. Disaccordance with previous reports that triploid CTCs are of no clinical significance in prognosis in gastric cancer (Li et al. 2016), our results showed pretreatment triploid CTCs (≥ 2/6 ml) were highly related to poor prognosis in advanced lung cancer patients, which may due to the differences in the biological characteristics of various cancer types. Therefore, in addition to small cell CTCs, the specific subpopulation of triploid CTCs is another key indicator in predicting poor prognosis. However, no positive relationship was established between the dynamic change of triploid CTCs number and patients’ treatment response.

Vim^+^ CTCs which represent distinct phenotype of EMT were detected and fully investigated in our study. Results revealed the presence of Vim^+^ CTCs in 19.7% of the included patients before chemotherapy administration. As for the quantitative composition, Vim^+^ CTCs accounted for 2.5% of the absolute CTCs number in pretreatment patients. Keeping in accordance with previous reports that tumor cells undergoing EMT were smaller in size than cells without EMT features (Ito et al. 2014; Ye et al. 2019), the majority of Vim^+^ CTCs detected in our study were in small size which may not be captured by cell size-based isolation technology but were efficiently isolated by SE-iFISH platform. Significant difference in cell size between Vim^+^ CTCs and vim^−^CTCs was found in our study, which implies not only cell surface makers but also cell size are in dynamic change during the process of EMT. Meanwhile, analysis was performed on Vim^+^ CTCs based on Chr8 ploidy and results revealed more than 80% of Vim^+^ CTCs were Chr8 aneuploidy. Clinical significance of both pretreatment and post-treatment Vim^+^ CTCs was investigated. The presence of Vim^+^ CTCs in pretreatment patients was correlated with liver metastases and meanwhile confirmed to significantly related to patient’ poor prognosis which was consistent with previous studies reported by others on prostate cancer studies (Satelli et al. 2017). Subgroup analysis suggested that the detection of Vim^+^ CTCs was of prognostic value in both SCLC and ADC patients. The prognostic role of Vim^+^ CTCs was further validated in multivariate analysis. After adjusting for clinically significant factors, baseline presence of Vim^+^ CTCs was the only independent predictor of PFS, which indicates Vim^+^ CTCs may serve as a marker for poor prognosis. Correlation of Vim^+^ CTCs (mostly in small cell size and Chr8 aneuploidy) to liver metastases and poor PFS in advanced lung cancer patients was demonstrated for the first time in our study. Clinical studies to further investigate how different cell sizes of aneuploid CTCs expressing different tumor biomarkers functionally cross-talk in cancer patients’ poor prognosis are currently conducted by us on a large cohort in lung cancer patients.

Our study has several limitations that must be acknowledged. First, this single center study included a relatively small number of patients for analysis, which may limit the statistical power of the research. Second, our study was based on two cohorts (SCLC cohort and ADC cohort) study according to pathological type which may induce the conclusions less comprehensive. Finally, the follow-up time was short and no OS data were available in this paper. Follow-up will be continued in future work.

In conclusion, our study demonstrated that CTCs from newly diagnosed advanced lung cancer patients were highly heterogeneous based on cell size, Chr8 aneuploidy and vimentin expression. Pretreatment small cell CTCs, triploid CTCs were potential predictive biomarkers for patients with advanced lung cancer. Baseline presence of Vim^+^ CTCs which were mostly in small cell size and Chr8 aneuploidy was associated with liver metastases and may serve as an independent predictor of poor PFS. These results may have implications in stratification of advanced lung cancer patients for personalized clinical management.

## Electronic supplementary material

Below is the link to the electronic supplementary material.

Supplementary material 1 (DOCX 12 kb)
