# Supplementary material for: Vimentin expression in circulating tumor cells (CTCs) associated with liver metastases predicts poor progression-free survival in patients with advanced lung cancer
Source: J Cancer Res Clin Oncol. 2019 Oct 23;145(12):2911–20. doi: 10.1007/s00432-019-03040-9 (PMC6861204; doi:10.1007/s00432-019-03040-9)
Supplement: Supplementary file 1 — Supplementary material 1 (DOCX 12 kb) [file 432_2019_3040_MOESM1_ESM.docx]

**Suppl Table1** Dynamic changes of CTCs subpopulations and treatment response.

| CTCs subpopulations | Treatment response | | *P* |
| --- | --- | --- | --- |
|  | PR | SD+PD |  |
| Total CTCs |  |  |  |
| Decrease+Stable | 23 | 7 | 0.024 |
| Increase | 14 | 15 |  |
| Small cell CTCs |  |  |  |
| Decrease+Stable | 26 | 9 | 0.026 |
| Increase | 11 | 13 |  |
| Large CTCs |  |  |  |
| Decrease+Stable | 19 | 9 | 0.437 |
| Increase | 18 | 13 |  |
| Triploid CTCs |  |  |  |
| Decrease+Stable | 24 | 11 | 0.261 |
| Increase | 13 | 11 |  |
| Tetraploid CTCs | 11 | 11 |  |
| Decrease+Stable | 26 | 11 | 0.119 |
| Increase | 22 | 11 |  |
| Multiploid CTCs | 15 | 11 |  |
| Decrease+Stable | 18 | 8 | 0.479 |
| Increase | 19 | 14 |  |
| Vim^+^CTCs |  |  |  |
| Decrease+Stable | 32 | 21 | 0.511 |
| Increase | 5 | 1 |  |
